# Comparative Volumetric Analysis of the Brain and Cerebrospinal Fluid in Chiari Type I Malformation Patients: A Morphological Study

**DOI:** 10.3390/brainsci9100260

**Published:** 2019-09-29

**Authors:** Seckin Aydin, Baris Ozoner

**Affiliations:** 1 Department of Neurosurgery, Okmeydani Training and Research Hospital, University of Health Sciences, Istanbul 34384, Turkey; 2 Department of Neurosurgery, School of Medicine, Erzincan Binali Yildirim University, Erzincan 24100, Turkey; barisozoner@gmail.com

**Keywords:** Chiari Type I malformation, volumetric morphometry, cerebrospinal fluid, gray matter, cognition

## Abstract

Background: Chiari Type I malformation (CM-I) is defined as the migration of cerebellar tonsils from the foramen magnum in the caudal direction and is characterized by the disproportion of the neural structures. The aim of this study was to investigate the brain volume differences between CM-I patients and normal population using a comparative volumetric analysis. Methods: 140 patients with CM-I and 140 age- and sex-matched healthy controls were included in this study. The magnetic resonance imaging (MRI) data of both groups were analyzed with an automated MRI brain morphometry system. Total intracranial, cerebrum, cerebellum, brainstem, cerebrospinal fluid (CSF), and lateral ventricle volumes as well as cerebrum and cerebellum gray/white matter (GM/WM) volumes were measured. Statistical analysis was performed. Results: Both total CSF and lateral ventricle volumes and volume percentages (Pct) were found significantly higher in CM-I patients compared to the control group. However, there were significant decreases in cerebrum and cerebellum volume Pct in CM-I patients. Although there were no significant differences in cerebrum WM volumes and volume Pct, cerebrum GM volume Pct were found to be significantly lower in CM-I patients. Conclusions: Revealing the increased CSF and lateral ventricle volume, and volume Pct supported concomitant ventricular enlargement and hydrocephalus in some CM-I patients. Decreased cerebrum GM volume Pct compared to the control group might be the underlying factor of some cortical dysfunctions in CM-I patients.

## 1. Introduction

Chiari type I malformation (CM-I) is defined as the migration of cerebellar tonsils from the foramen magnum in the caudal direction [1,2,3], and the widely accepted limit value of this migration is 5 mm and above in clinical trials [4,5]. There are several theories to explain the pathophysiology of this entity such as developmental arrest theory, hydrodynamic theory, and small posterior fossa/hindbrain overgrowth theory [6,7,8,9,10,11].

So far, various morphometric studies have been made to reveal the dynamics of posterior fossa, cerebellum, and cerebrospinal fluid (CSF) in patients with CM-I [2,11,12,13,14,15,16]. However, these studies are usually based on linear calculations of radiological data and morphometric volume calculations using planimetric or point counting techniques. Recently, it has become possible to calculate the gray/white matter (GM/WM) and CSF volumes in several brain pathologies using volumetric morphometry techniques with advanced computer applications [17,18,19,20,21,22].

CM-I is thought to be characterized by the disproportion of the neural structures, and CM-I patients are considered to have differences in terms of the volumes of intracranial components compared to the normal population. The aim of this study was to investigate the brain GM/WM and CSF volume differences between CM-I patients and normal population using a comparative volumetric analysis.

## 2. Materials and Methods

### 2.1. Patients and Control Subjects 

After the approval of the Erzincan Binali Yildirim University ethics committee, magnetic resonance imaging (MRI) records were examined between 2014 and 2018. Of 178 patients with CM-I, 140 were included in the study. A total of 38 patients were excluded due to insufficient clinical and radiological data. All 140 patients had 5 mm or more downward herniation of the cerebellar tonsils from foramen magnum. In addition, all patients had a history of hospital admission with CM-I symptoms (valsalva-induced suboccipital head and neck pain, numbness or paresthesias in extremities, etc.). While 34 patients had syringomyelia in various sizes, none of them had hydrocephalus. Patients with a previous history of operation for CM-I, hydrocephalus or syringomyelia; those with congenital skull base anomalies (platybasia etc.), intracranial occupying mass lesion or spinal pathology; and those under 18 years of age were excluded from the study. 

To assure the correct interpretation of statistical inferences, the control group was composed of subjects with exactly the same age and gender as the CM-I group. In other words, the subjects of the control group had no tonsillar herniation or history of neurological or psychiatric disorders or symptoms in their medical records.

### 2.2. Imaging and Morphometrical Data Collection

Magnetic resonance imaging (MRI) was performed for both groups using a 1.5 Tesla MR (SignaHDxt; 1.5 Tesla; General Electric, Boston, MA, USA) scanner with 3 mm thickness and a standard head coil. Assessments were performed on the T1-weighted axial sections.

**Figure 1:**
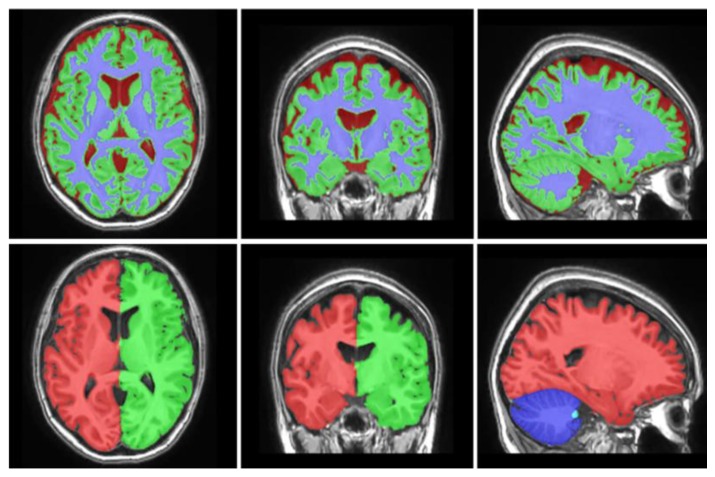
Brain gray and white matter discrimination and macrostructures with automated magnetic resonance imaging (MRI) brain morphometry.

Data were analyzed with an automated MRI brain morphometry system based on multi-atlas label fusion technology that was previously used and validated in Chiari Type I malformation patients (Figure 1) [23,24]. The method we chose was available and freely accessible through the online web interface [25].

Collected MRIs of the patients were stored in a zipped file, and then the data was uploaded to the website online. Thereafter, absolute values were obtained for more than 20 parameters, and we used 13 of these to investigate the volumetric information.

Intracranial volumes could be affected by age, sex, and physical characteristics, therefore, we preferred to use the volume percentages in order to obtain more accurate results. So, volume measurements (cm^3^) and volume percentages (Pct) (%—the ratio of subject/total volume) were considered as two distinct variables. In this study, total intracranial, total cerebrum, cerebrum GM/WM, total cerebellum, cerebellum GM/WM, brainstem, CSF, and lateral ventricle volumes/volume Pct of both the CM-I and control groups were measured.

### 2.3. Statistical Analysis

Statistical analysis was performed using the IBM SPSS version 19 package program (IBM Software, New York, NY, USA). The results for continuous variables are provided as mean ± standard deviation. Normality of distribution for continuous variables was evaluated using the Kolmogorov–Smirnov test. Depending on whether the statistical hypotheses were fulfilled, either the Student's *t*-test or the Mann–Whitney *U* test was used to compare the independent continuous variables between the two groups. Holm–Bonferroni Method was used to deal with familywise error rates (FWER) for multiple hypothesis testing. A *p* value < 0.05 was considered statistically significant.

## 3. Results

As both groups were composed of subjects at the same age and gender, the mean age and gender distribution of the two groups were the same. Therefore, there were 105 (75%) women and 35 (25%) men in both groups. The mean age was 39.4 ± 11.9 years (range, 18–69 years).

There were no significant differences between the groups in terms of intracranial (*p* = 1.000), cerebrum (*p* = 1.000), cerebellum (*p* = 0.657), and brainstem (*p* = 1.000) volumes. However, cerebrum (*p* = 0.052) and cerebellum (*p* = 0.002) volume Pct were found to be significantly lower in CM-1 patients (Table 1).

**Figure 2:**
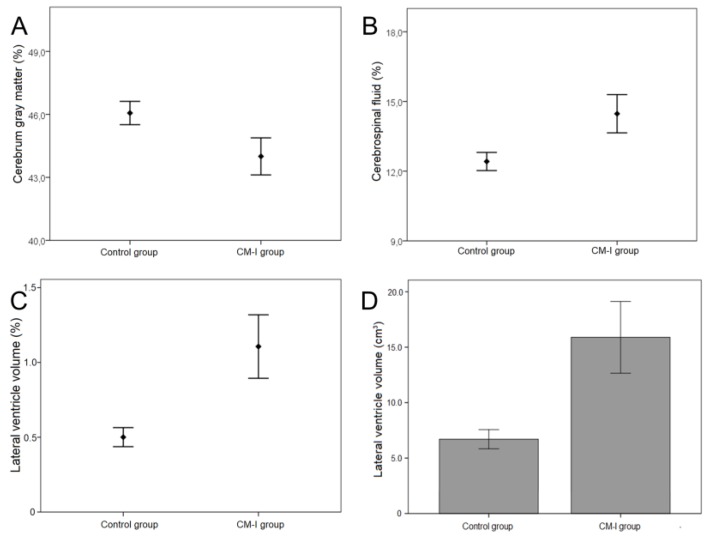
(**A**) The statistical differences of cerebrum gray matter volume percentages (%) between CM-I group (44.0 ± 5.3) and control group (46.1 ± 3.3) are shown (*p* = 0.002). (**B**) The statistical differences of cerebrospinal fluid volume percentages (%) between CM-I group (14.5 ± 4.9) and control (12.4 ± 2.4) are shown (*p* = 0.002). (**C**) The statistical differences of lateral ventricle volume percentages (%) between CM-I group (1.1 ± 1.3) and control group (0.5 ± 0.4) are shown (*p* = 0.002). (**D**) The statistical differences of lateral ventricle volume (cm^3^) between CM-I group (15.9 ± 19.4) and control group (6.7 ± 5.2) are shown (*p* = 0.002).

There was a statistically significant increase in both total CSF and lateral ventricle volumes (*p* = 0.002) and volume Pct (*p* = 0.002) in CM-I group when compared to the control group (Figure 2B–D, Table 2).

There were no significant differences in terms of cerebellum GM (*p* = 1.000) and WM (*p* = 0.672) volumes, and GM (*p* = 0.253) and WM (*p* = 0.228) volume Pct in the CM-I group compared to the control group. Although there were no significant differences in terms of cerebrum WM volume (*p* = 1.000) and volume Pct (*p* = 1.000), cerebrum GM volume Pct (*p* = 0.002) were found to be lower in the CM-I group (Figure 2A, Table 3). 

## 4. Discussion

So far, several morphometric studies have been reported for CM-I patients about posterior cranial fossa, cerebellum, and intracranial volume or CSF dynamics compared to the normal population [1,2,9,10,11,23,24]. While some of the parameters (intracranial measurements, cerebrum, or CSF volume, etc.) have been studied in previous morphometric studies, others (cerebrum and cerebellum GM/WM volume, etc.) have not been used before to the best of our knowledge. In this study, we aimed to investigate the brain volumes of CM-I patients with a sensitive computer-based method to compare them with the normal population. 

As the first inference, cerebellum total volume Pct was found lower in CM-I patients compared to the control group. In the literature, posterior cranial fossa volume (PCFV) is usually measured instead of the cerebellum because of technical limitations [2,23,24,26]. The results gathered from these studies generally indicate that there is no significant difference in PCFV compared to the normal population. However, in these studies, the brainstem and cerebellum volumes were included in the PCFV, and morphometric data of these structures were reported together. In the morphometry technique we applied, brainstem and cerebellum volumes were examined separately and, as we mentioned above, the cerebellum volume Pct was lower than the control group. However, no significant difference was observed in brainstem volume Pct. Besides, these findings do not contradict the theory of the small posterior fossa, it shows that the cerebellum volume percentage is more affected than the brain stem because of the compression of the posterior fossa.

In the literature, the total intracranial volume (ICV) was measured in CM-I patients and was generally used for comparison with PCFV, so far [13,27]. In our study, no significant difference was found between the control group and the CM-I group in terms of total ICV. However, different results were obtained when brain tissue and CSF volumes were measured separately due to the design of this study.

In our study, we found a statistically significant increase in total CSF and lateral ventricle volume and volume Pct in CM-I group compared to the control group. The relationship between ventricular expansion or hydrocephalus and CM-I have long been known, and its incidence was found to be 10% in some series [10,28,29]. Several theories have been proposed to explain the pathogenesis of concomitant hydrocephalus in CM-I patients. According to these, the impairment of CSF hydrodynamics could be originated from the overcrowding of posterior fossa neural structures [30,31], posterior fossa hypoplasia [32], jugular foramen stenosis [33], and cerebellum overdevelopment in some congenital syndromes [34,35]. In addition, the abnormal intraventricular CSF pressure could be induced by anatomical obstruction of pericerebellar spaces, functional obstruction of the foramen Magendie [36] or an anatomo-functional obstruction at the level of foramen magnum [37,38]. In a comprehensive study, Di Rocco et al. stated that CM-I and ventricular dilatation were related to multiple heterogeneous factors, this is called multifactorial pathogenetic theory [39]. On the other hand, Milhorat et al. claimed that syringomyelia and hydrocephalus develops secondary to chronic tonsillar herniation [10]. In this study, none of the patients had a history of hydrocephalus surgery or clinical signs of hydrocephalus at the time of admission. So, we can consider that the significant lateral ventricle enlargement in this group of patients could be accepted as an early stage of the hydrocephalus development process or an abnormal ventricle enlargement due to various pathogenetic mechanisms mentioned above. Due to limitations of this study, further detailed information could not be obtained from patients. Future prospective studies, including clinical follow-up with neuropsychiatric examinations, are needed to achieve more comprehensive understanding of pathophysiology.

Perhaps one of the most striking results in our study was the significant reduction in the cerebrum GM volume Pct in CM-I patients compared to the control group. It was difficult to explain the underlying mechanism of this finding at first, because no similar study has been conducted in CM-I patients in the literature. Based on the relationship between CM-I and hydrocephalus, we initially reviewed the literature in terms of hydrocephalus. Several mechanisms have been asserted in the pathogenesis of GM reduction due to hydrocephalus. In the past, experimental and human congenital hydrocephalus studies have shown the enlargement of the periventricular subependymal space and extracellular space [40,41,42,43]. Hence, hydrostatic forces and biochemical changes induced by CSF collection in the extracellular space and stretching of the ventricle enlargement cause damage to the GM [44]. In addition, several experimental animal studies reported the decrease of cortical GM with the increase of the ventriculomegaly [45,46,47]. Fletcher et al. reported that the increase of CSF in patients with hydrocephalus interestingly led the decrease in GM Pct, in contrast to WM Pct [48]. In a study reported by Zhang et al., cortical thickness and WM integrity of hydrocephalus patients were measured by radiological methods, and a significant decrease of temporal and frontal lobe cortical GM thickness, as well as a decrease in corpus callosum WM fraction anisotropy were observed in hydrocephalus patients [49]. According to Monro Kellie doctrine, it is also possible that the increase in the amount of CSF may lead to a decrease in the brain parenchyma [50]. As the cortical GM is in contact with the calvarial bone, theoretically, GM is reduced due to an increase in intrinsic pressure. This study is basically based on the hypothesis that investigates volume changes in CM-I patients. It is a matter of curiosity whether the decrease in GM Pct reported in CM-I patients might be explaining some clinical findings. Several studies have reported that there was a certain relationship between some neuropsychiatric disorders and GM volume reduction in specific brain regions [51,52,53,54,55]. Some authors have claimed that neurocognitive disorders in CM-I patients cannot be explained only by cerebellar involvement, and they stated that cerebellar and cerebral connections may play a role in these disorders [56,57]. Although our findings might explain cognitive disturbances in some CM-I patients, the understanding of the etiopathogenesis of some cortical dysfunctions in CM-I patients is still incomplete. 

Since Chiari is not a pathology affecting a specific cerebral region, we performed a total volumetric analysis rather than a segmental volumetric analysis. Therefore, while evaluating the cerebrum GM reduction in Chiari patients, it’s not possible to determine the exact affected cortical region. As the first study in this field, it is appropriate to consider this research as a preliminary study. Our results must be validated by further morphometric studies, and segmental brain GM volume analysis should be performed in CM-I patients.

## 5. Conclusions

This morphometric study was conducted with the hypothesis that Chiari malformation is a disorganization in neural structures. Thus, we measured the brain and CSF volumes of the CM-I group and compared them with a control group. First, we found an increased CSF and lateral ventricle volume and volume Pct, which supports the accompanying abnormal ventricle enlargement in CM-I. Subsequently, we revealed a decreased cerebrum GM volume Pct that might be the underlying factor of some cognitive dysfunctions in CM-I patients. Further morphometric studies on segmental cortical GM will provide more detailed information to complement our data.

## Figures and Tables

**Table 1:** Comparison of total intracranial, cerebrum, cerebellum, and brainstem volumes and volume percentages between Chiari type I malformation (CM-I) and control patients.

Variables	CM-I	Control	Mean Difference	95% Cl	*p* Value *
Total ICV (cm^3^)	1344.6 ± 133.3	1328.9 ± 116.8	−15.69	−45.17 to 13.79	1.000
Cerebrum Volume (cm^3^)	1004.3 ± 119.3	1013.9 ± 96.0	9.66	−15.81 to 35.13	1.000
Cerebrum Volume Pct (%)	74.6 ± 4.4	76.3 ± 2.2	1.64	0.82 to 2.47	0.052
Cerebellum Volume (cm^3^)	130.8 ± 74.3	128.4 ± 11.9	−2.39	−14.91 to 10.14	0.657
Cerebellum Volume Pct (%)	9.3 ± 0.9	9.7 ± 0.6	0.41	0.23 to 0.58	0.002
Brainstem Volume (cm^3^)	21.7 ± 3.4	21.6 ± 2.3	−0.18	−0.87 to 0.51	1.000
Brainstem Volume Pct (%)	1.6 ± 0.2	1.6 ± 0.1	0.00	−0.05 to 0.04	1.000

Data are presented as mean ± standard deviation. CM-I—Chiari Type I malformation; CI—Confidence interval; ICV—Intracranial volume; Pct—Percentage. * Significance of differences in comparisons between the patient cohorts was determined using the Holms–Bonferroni method.

**Table 2:** Comparison of total cerebrospinal fluid and lateral ventricle volumes and volume percentages between CM-I and control patients.

Variable	CM-I	Control	Mean Difference	95% Cl	*p* Value *
Total CSF Volume (cm^3^)	194.2 ± 66.5	165.1 ± 35.2	−29.12	−41.64 to −16.59	0.002
Total CSF Pct (%)	14.5 ± 4.9	12.4 ± 2.4	−2.05	−2.96 to −1.15	0.002
Lateral ventricle Volume (cm^3^)	15.9 ± 19.4	6.7 ± 5.2	−9.24	−12.58 to −5.91	0.002
Lateral ventricle Pct (%)	1.1 ± 1.3	0.5 ± 0.4	−0.61	−0.83 to −0.39	0.002

Data are presented as mean ± standard deviation. CM-I—Chiari Type I malformation; CI—Confidence interval; CSF—Cerebrospinal fluid; Pct—Percentage. * Significance of differences in comparisons between the patient cohorts was determined using the Holms–Bonferroni method.

**Table 3:** Comparison of cerebrum and cerebellum gray/white matter volumes and volume percentages between CM-I and control patients.

Variables	CM-I	Control	Mean Difference	95% Cl	*p* Value *
Cerebrum GM Volume (cm^3^)	592.2 ± 94.3	612.3 ± 71.2	20.13	0.48 to 39.79	0.450
Cerebrum GM Volume Pct (%)	44.0 ± 5.3	46.1 ± 3.3	2.06	1.03 to 3.10	0.002
Cerebrum WM Volume (cm^3^)	412.1 ± 64.9	401.6 ± 53.1	−10.47	−24.43 to 3.48	1.000
Cerebrum WM Volume Pct (%)	30.6 ± 3.7	30.2 ± 3.0	−0.42	−1.22 to 0.37	1.000
Cerebellum GM Volume (cm^3^)	88.4 ± 15.0	90.6 ± 12.0	2.19	−0.99 to 5.38	1.000
Cerebellum GM Volume Pct (%)	6.6 ± 1.0	6.8 ± 0.8	0.24	0.03 to 0.45	0.253
Cerebellum WM Volume (cm^3^)	36.1 ± 8.5	37.8 ± 8.1	1.76	−0.18 to 3.71	0.672
Cerebellum WM Volume Pct (%)	2.7 ± 0.6	2.9 ± 0.6	0.17	0.03 to 0.31	0.228

Data are presented as mean ± standard deviation. CM-I—Chiari Type I malformation; CI—Confidence interval; GM—Gray matter; WM—White matter; Pct—Percentage. * Significance of differences in comparisons between the patient cohorts was determined using the Holms–Bonferroni method.
